# Supplementary material for: Are carbon emissions trading and green financial instruments synergistic? -Comprehensive quantitative research based on content analysis
Source: PLoS One. 2024 Mar 7;19(3):e0298601. doi: 10.1371/journal.pone.0298601 (PMC10919601; doi:10.1371/journal.pone.0298601)
Supplement: S1 Data — (ZIP) [file pone.0298601.s001.zip › Data/地级市Z_F工作报告.zip!/╦─┤¿/┤∩╓▌╩╨/└┤╫╘╣1⁄2╓┌║┼í╛┬φ┐╦╩2╛▌═°í┐_2017.docx]

各位代表：

我代表市人民政府向大会报告工作，请予审查，并请市政协委员提出意见。

一、2016年工作回顾

过去一年，面对错综复杂的宏观经济形势、达州发展中的特殊困难和经济下行压力持续加大的严峻挑战，在省委省政府和市委的坚强领导下，市人民政府团结带领并紧紧依靠全市人民，克难攻坚、负重前行，取得了来之不易、令人鼓舞的可喜成绩。全年实现地区生产总值1447.1亿元、增长7.5%，地方一般公共预算收入84.7亿元、增长8.5%，固定资产投资1500.1亿元、增长12.6%，社会消费品零售总额761.7亿元、增长13.3%，城镇居民人均可支配收入26016元、增长8.9%，农村居民人均可支配收入11718元、增长9.6%，圆满完成了市三届人大七次会议确定的目标任务，实现了“十三五”良好开局。回顾过去一年，我们着重开展了以下8个方面的工作：

精准施策稳增长，经济实现止滑回升。狠抓工业稳增长，一企一策帮助企业解决具体困难，普光净化厂缩短检修时间并持续稳产，南坝净化厂、川投燃机发电等44个项目竣工投产，75户企业升规入统，规上工业增加值增长7.6%，同比提高10.6个百分点。狠抓服务业稳增长，着力解决漏项短项缺项问题，清理规上企业入库118户，培育“小升规”企业98户，督促外地建筑和房地产企业在达登记注册11家，服务业增加值增长9.3%，高于全省0.2个百分点。狠抓农业稳增长，增加值增速达4.1%。狠抓投资稳增长，大力开展“项目攻坚年”活动，集中力量推进30个重大项目和200个重点项目建设，重点项目完成投资483亿元。狠抓消费稳增长，积极参加全省市场拓展“三大活动”〔1〕，大力开展各类促销，消费对经济增长的贡献率达58%。

坚定不移调结构，转型发展步伐加快。力促工业转型升级，制定实施了转型升级意见和年度工作方案，重点推进了瓮福集团等18户企业转型升级，启动实施了川东电缆等40个技改项目，正原微玻纤二期、新达泵阀、曦鸿电子等项目建成投产，悦安华川汽车新厂区开工建设，安凯清洁能源汽车总装车间竣工。力促服务业提档升级，秦巴物流园区、仁和春天等项目加快建设，红星美凯龙、罗浮城市广场等项目建成运营，服务业完成投资近1000亿元；巴山大峡谷开工建设，新增国家4A级景区4个，旅游总收入140.5亿元、增长25%；电子商务加快发展，实现交易额86.5亿元、增长41%。力促现代农业发展，新建农建综合示范区21万亩、高标准农田15.5万亩、特色产业基地10.4万亩，新培育市级以上龙头企业21家。力促建筑业和房地产、现代金融、农产品加工和贸易等优势潜力产业发展，建筑业、房地产业增加值分别增长11.7%和5%，金融业增加值增长18.3%，农产品加工和贸易收入突破200亿元。

攻坚克难强基础，城乡面貌明显改变。加快推进重大基础设施建设，南大梁高速全线贯通，达州新机场、巴万高速、土溪口水库开工建设，营达高速、达宣达开快速通道、6座中型水库加快建设，新改扩建国省干道52.8公里、农村公路1158公里，农网升级改造和宽带乡村工程加快实施。突出抓好中心城区建设，“五桥六路”〔2〕“七大新区”〔3〕布局展开，凤凰山隧道、金南大桥、环城路二期等加快建设，新增建成区面积6.5平方公里、人口5万人，“双百”区域中心城市〔4〕加快形成；大力实施“缓堵保畅”和“五治”“三化”工程〔5〕，打通城区断头路7条，新建停车泊位1.1万个，中心城区PM10、PM2.5分别下降13.7%和8.9%，塔沱片区等旧城改造加快实施，徐家坝收费站改建、塔沱滨河公园、鹿鼎寨公园、莲花湖休闲步道等建成投用，重点街区和道路实现亮化美化，城市品质进一步提升。市云计算中心启动实施，智慧城市加快建设。统筹城镇和新村建设，大竹、渠县撤县设市顺利推进，5个县城规模不断扩大；全域纳入国家新型城镇化试点，宣汉南坝入选国家首批特色小城镇，撤乡设镇14个，全市城镇化率提高1.55个百分点；新建幸福美丽新村300个。

千方百计增动能，改革创新不断深化。推进供给侧结构性改革，保优汰劣去钢铁产能140万吨、煤炭产能105万吨，多措并举去房地产库存57万平方米，降低企业用电用气成本1.26亿元，落实减税降费减轻企业负担10亿元。推进“4+2”重点改革〔6〕，城市公交体制改革顺利完成，市区责权体制、环卫市场化管理、城市建管分离改革全面展开，行政效能、目标绩效考核不断优化。推进国企改革，34户市属停破产国企改革全面完成，10类26户国企改革激活方案启动实施。推进农业农村改革，土地确权登记全面完成。深化财税和投融资体制改革，“营改增”试点全面推开，争取地方政府债券179亿元、国家专项建设基金88亿元，推广PPP引进社会资本115亿元，川环科技成功上市。大力实施创新驱动战略，制定出台了科技创新“十个一批”〔7〕措施，组建设立了微玻纤研究院和智能制造学院，创建国家高新技术企业6家、市级以上企业技术中心8家，科技对经济增长的贡献率达50%；落实大众创业、万众创新“20条措施”，新登记市场主体2.4万户。

借势借力激活力，开放合作成果丰硕。大力开展制造业招商，先后举办了电子信息、精细化工等产业招商洽谈会，全年引进项目156个，到位资金668.9亿元。着眼产业发展需要，切实加强与国内外、央企省企、银政银企、校地校企等深度合作，中捷产业园、玄武岩纤维开发、磷酸盐产业园、铁山旅游综合开发等项目正在深入洽谈和合作，与北京信托、成都农商银行等金融机构签订多项合作协议，成功获得政策性银行贷款29亿元，与清华大学开展了人才培养和引进合作。大力发展外经外贸，新增外商投资企业2家、海关备案企业21家，合同利用外资1.1亿美元，完成进出口总额1.6亿美元。

众志成城补短板，脱贫攻坚首战告捷。坚持把脱贫攻坚作为头等大事，举全市之力打好开局之战，149个贫困村实现退出、12.77万人实现脱贫，年度目标全面完成。认真落实“六个精准”〔8〕“六个一批”〔9〕，全面对标“两不愁”“三保障”“四个好”〔10〕，完善了脱贫攻坚规划，锁定了决胜目标。明确党政“双组长”负责制，制定实施八项超常措施，压实了工作责任。创新开展“5+1”帮扶机制〔11〕，贫困村驻村帮扶实现全覆盖。开展“万企帮万村”行动，协调127家企业结对帮扶了130个贫困村。改善贫困群众住房条件，完成易地扶贫搬迁3.1万人，改造贫困户危房1.1万户。加强基础设施建设，脱贫村通村公路硬化和脱贫人口安全饮水、用电等问题得到解决。加快产业发展助农增收，整合各类涉农资金59.9亿元，市县共建扶贫基金11.7亿元，帮助5.8万扶持对象落实了产业项目。加强教育和医疗扶贫，减免贫困家庭学生费用3.4亿元，落实医疗“十免四补助”〔12〕6965万元。

坚持不懈惠民生，人民生活持续改善。各项民生投入255亿元，占公共财政支出的70.9%。认真办理民生实事，九项民生工程、17件民生大事全面兑现，改造棚户区及农村危房3.7万户，城镇新增就业4.3万人，五项社会保险参保覆盖516万人次，新增养老床位2560张。加强生态环境保护，启动了绿化达州行动，完成营造林29.6万亩，新增森林6322公顷，治理水土流失131平方公里，主要河流出口断面水质稳定在Ⅲ类标准。加快社会事业发展，新改扩建中小学校109所，主城区达州嘉祥等6所学校建成投用，通川区义务教育均衡发展通过国家认定，达州职业技术学院部分搬迁入园；深入实施文化惠民工程，市图书馆、规划馆、工青妇活动中心等建成开放；医疗卫生综合改革不断深化，重大传染病防治切实加强，与华西医院合作办医深入推进；全民健身活动广泛开展，我市体育健儿首次夺得残奥会冠军，成功举办市第三届运动会和残运会。

持之以恒转作风，自身建设切实加强。讲政治守规矩，深入践行“三严三实”，扎实开展“两学一做”，严格执行中央八项规定精神，认真落实省、市委相关规定，“三公”经费下降20%；切实加强政府机关干部的教育管理，坚定地维护风清气正的政治生态。大力推进依法行政，自觉接受市人大的法律监督、工作监督和市政协的民主监督，广泛听取并采纳各方意见建议，法治政府加快建设。深入推进“放管服”改革〔13〕，取消行政许可事项34项。扎实推进“法律七进”〔14〕，强化社会治安综合治理，依法打击各类违法犯罪，社会保持和谐稳定。切实加强食品药品和安全生产监管，连续三年未发生重特大事故。严格落实党风廉政建设责任制，加强行政监察、审计监督，正风肃纪持续深化。

同时，我们扎实推进国防动员、人民防空、民兵预备役、交通战备、双拥和优抚安置工作，成功创建全国双拥模范城。积极做好民族、宗教、对台、外事、侨务、文联、科协、统计、档案、保密、气象、地方志等工作，大力支持妇女、儿童、老龄、残疾人、慈善、红十字会等事业发展，各方面工作都取得了新的进步。

各位代表！过去一年取得的成绩，是省委省政府和市委正确领导的结果，是市人大和市政协有效监督、大力支持的结果，是全市干部群众不甘落后、锐意进取的结果。在此，我代表市人民政府向全市人民，向人大代表、政协委员，向各民主党派、工商联、无党派人士和人民团体，向驻达解放军指战员、武警官兵和政法干警，向所有关心支持达州建设发展的社会各界人士，表示衷心的感谢！

各位代表！在看到成绩的同时，我们也清醒认识到，人口多、底子薄、欠发达、不平衡的基本市情尚未根本改变，全市改革发展还面临不少问题和挑战：部分传统产业优势减弱，新兴产业处于孕育期，企业融资难融资贵仍然存在，经济企稳向好的基础还不牢固，下行压力依然较大；产业结构性矛盾突出，企业主体创新能力不强，产品低端化特征明显，转型发展还任重道远；贫困人口面广量大，城乡基础设施和公共服务欠账较多，环境保护、教育医疗、食品药品监管、城市管理等与群众的期望还有较大差距，脱贫攻坚、民生改善任务还十分艰巨；少数政府工作人员不廉不勤等问题还不同程度存在，依法行政水平和政务服务效能还有待进一步提高等等。对此，我们一定高度重视，采取更加有力措施不断加以解决。

二、2017年目标任务

今年是市政府换届后的第一年，是实施“十三五”规划的重要一年，也是推进供给侧结构性改革的深化之年，我们将迎来党的十九大和省第十一次党代会召开，做好各方面工作至关重要。今年政府工作的总体要求是：深入贯彻以习近平同志为核心的党中央治国理政新理念新思想新战略，认真落实市第四次党代会和市委四届二次全会暨经济工作会议部署，始终保持专注发展、转型发展定力，自觉适应把握引领经济发展新常态，坚持稳中求进工作总基调，坚持以提高发展质量和效益为中心，坚持以推进供给侧结构性改革为主线，突出加快发展、负重转型、追赶进位工作取向，更加注重决胜脱贫攻坚，更加注重产业转型升级，更加注重振兴实体经济，更加注重全面改革创新，统筹抓好稳增长、促改革、调结构、惠民生、防风险各项工作，确保全市经济平稳健康发展、社会和谐稳定。

今年全市发展的主要预期目标是：地区生产总值增长7.5%，全社会固定资产投资增长10%，社会消费品零售总额增长12%，地方一般公共预算收入增长7.5%，城镇和农村居民人均可支配收入分别增长8.5%和9.5%，居民消费价格涨幅控制在3%左右，城镇登记失业率控制在4.2%以内，节能减排和环境保护完成省政府下达的目标。在实际工作中，我们将按照以下要求，全力以赴争取更大业绩。

——坚持稳中求进。稳中求进是治国理政的重要原则，是做好经济工作的方法论。针对当前经济稳定增长基础不牢、后劲不足等问题，我们将把稳增长作为主要矛盾，精准施策，全力以赴稳产业、稳投资、稳消费，防范经济增长大起大落。在稳的基础上，我们将着力坚持扩总量、提速度与提质量并举，以进促稳，以积极的姿态跳起摸高谋求更大发展，以较高增速在发展中、运动中求稳。全年经济工作将按明显高于预期指标来布置落实。

——坚持提质增效。提质增效是经济转型升级的必然要求，是达州持续发展的紧迫需要。我们将更加注重工业转型升级，积极引导企业通过技术改造、开放重组等方式增强传统产业动力，激活存量；千方百计发展新材料、智能制造、节能环保装备等新兴产业，加快形成新的经济增长点；全面推进服务业提档升级，加快三大优势潜力产业发展，努力提高企业经济效益，大力培育税源财源，不断改善提升财政收入结构和质量。

——坚持改革创新。改革创新是市委提出的重大战略，是达州后发追赶的根本动力。我们将抓住四川推进全面创新改革试验的机遇，不断建立健全有利于达州全面创新和改革发展的体制机制。坚持问题导向，把改革创新贯穿到政府工作的全过程，全面推进有利于促进发展、改善民生、激发积极性创造性的改革。坚持向创新要质量要效益要速度，支持企业开展制度创新、管理创新、技术创新，依靠创新驱动打造发展新引擎。

——坚持发展为民。发展为民是践行共享发展的根本要求，是政府工作的根本宗旨。我们将坚持把脱贫攻坚作为头等大事和第一民生工程，精准发力、精准施策，不折不扣完成好年度目标任务。持续抓好民生工程、办好民生实事，让全市广大人民群众得到更多的实惠、有更多的获得感。继续从解决群众最关心最直接最现实的利益问题入手，做好普惠性、基础性、兜底性民生建设，全面提高公共服务水平，进一步织密扎牢民生保障网。

各位代表！上述目标，既充分考虑了与全国全省同步全面小康、稳定市场信心和增加就业的需要，也充分考虑了宏观经济环境和现实支撑条件，既与中央和省委的部署相衔接，又与全市“追赶进位”的要求相匹配，只要我们咬定青山不放松，面对困难不退缩，团结奋斗不懈怠，就一定能够圆满完成既定的目标任务！

三、2017年重点工作

今年是我市经济恢复性增长经受考验、脱贫攻坚进入关键阶段的一年，我们将按照市第四次党代会“12335”总体部署〔15〕和四届人大一次会议批准的政府工作报告“681”目标任务〔16〕，突出重点、攻坚克难，努力开创全市改革发展新局面。

（一）系统推进工业转型升级。我市稳增长调结构的重点在工业、关键在工业。我们将认真落实工业转型升级意见，细化目标、系统推进，确保规上工业增加值增长7.8%以上。

聚焦产业集群培育。加快促进中石油铁山坡、麻柳天然气新井勘探开发，抓好精细化工产业园、天然气分布式能源等项目建设，培育能源化工“千亿产业集群”。加快发展微玻纤、玄武岩纤维“两大纤维”及其制品，抓好安凯清洁能源汽车、悦安华川特种车“两车”项目建设，大力发展汽车零配件、健康家电、五金工模具、节能环保装备、智能仪表等产业，培育新材料和智能装备制造两个“百亿产业集群”。积极引进一批龙头企业，大力发展电子信息、农产品加工、生物制药等产业，培育一批“超十亿产业集群”。围绕重点产业集群发展，实施重点项目落地计划，力争工业招商落地项目100个、到位资金380亿元，开工建设重点项目100个、完成工业投资420亿元，实施重点技改项目100个、完成技改投资295亿元。启动建设建筑产业园，新增建筑资质企业20户，确保建筑业增加值增长13%以上，助力第二产业发展。

聚焦企业和产品培育。实施“五十百千”〔17〕企业培育工程，重点推动达钢转型发展和普光净化厂满负荷生产，确保2个百亿企业不掉队；加快磷酸盐产业园建设，力促瓮福化工向五十亿级企业迈进；加强西部鞋服、川投燃机发电、正源微玻纤、川环科技、新达泵阀、金恒机械、富士电梯、乐仕达电子等企业培育，新增超十亿元企业2户；做大做强鹏翔电子、立川食品、琦云药业、达欧机械等企业，新增超亿元企业20户；加快推进“成长型”“小升规”企业发展，新培育超千万元企业250户，新增升规入统企业50户。整合优化工业存量，加快冶金、建材、化工、食品等传统产业升级改造，支持企业通过制度创新、管理创新、技术创新、产品创新、对外合作等方式激发活力，推动结构、质量和效益大提升，新培育示范企业20户，开发新产品20个以上。

聚焦产业园区建设。加强“1+7”园区基础设施和服务平台建设，促进产城融合、园区互动发展，提升投资吸引力、项目承载力和产业竞争力。加快园区污水处理等配套设施建设，降低园区要素成本。引导优势企业向园区集聚，抓好已建“标准厂房”的招商入驻，提升产业关联度和集聚度。加强对园区“围而不建、建而不产、建成而又停产多年”的项目和企业清理，倒逼项目落实落地和企业转型升级。着手推进“市农产品加工集中区—达川商贸物流园区—达州经开区—达川工业园区—达州空港新区—大竹工业园区”产业带建设。大力支持园区创建国家和省级经开区、高新区。

（二）全面推进服务业提档升级。服务业是我市经济、财税和就业的重要支撑，我们将继续在强基础、扩规模、上档次、提质量、创品牌、优环境上狠下功夫，确保服务业增加值增长9%以上。

加快重点领域发展。突出发展现代金融业，全面落实促进金融业发展“十条”意见，抓好马踏洞金融集聚区建设，推动市商业银行异地设立分支机构，新增社会融资250亿元，确保金融业增加值增长16%以上。大力发展现代物流业，抓好秦巴物流园区、物流信息平台、物流联盟“三个龙头”，带动“九大体系”〔18〕建设，打造秦巴地区“无水港”。创新发展现代商贸业，抓好通川复兴、达川杨柳等商贸集聚区和仁和春天、升华广场等城市综合体建设，千方百计将消费留在达州。着力发展旅游业，开展“三年攻坚行动”，办好市第六届旅游业发展大会，抓好八台山—巴山大峡谷、铁山—莲花湖—凤凰山—双鱼湖等片区综合开发，新增国家4A级景区4个、3A级2个和省级生态旅游示范区1个，实现旅游总收入170亿元。加快发展电子商务，抓好电商集聚区和示范县（区）建设，做实做大做强电商企业，重点解决“买多卖少”“异地企业注册”问题，力争交易额增长45%。支持邮政和快递业发展，加快川东北快递分拨处理中心建设。加强房地产市场的调控和引导，促进行业平稳健康发展。

着力提升品牌质量。抢抓全省实施服务业“三百工程”〔19〕的契机，加强对企业创牌引导和培训，支持一批优势企业创著名商标、创星级酒店、创4A和5A级景区等品牌，鼓励其制定行业和产业标准，在做响品牌、输出服务中占据主动。继续抓好大市场、大卖场的改造提升，引导和支持企业运用高新技术、信息技术加强现代化和网络化管理。积极适应消费升级，加强服务业供给侧结构性改革，大力发展以互联网为载体、线上线下互动的服务业新业态，着力增强现代服务业发展活力。

扎实做好基础工作。加大服务业投入力度，抓好“十亿级”重点项目建设，力争服务业完成投资1100亿元。加强重点企业培育和引进，新增“小升规”企业100户、限上企业30户。认真落实促进服务业发展的各项政策，加大土地使用、信贷融资、技术创新、市场开拓等扶持力度。完善政府、企业、媒体联动的整体宣传推广机制，扩大服务业在区域中的影响力和知名度。建立服务业发展统筹推进机制，继续抓好漏项短项缺项问题的解决，强力推进外地在达从事房地产开发企业、大型连锁企业分支机构在达登记注册独立法人，防止产值漏统、税收流失。

（三）加快推进现代农业发展。发展现代农业是我市由农业大市向农业强市转变的必然选择，我们将大力推进农业供给侧结构性改革，着力提高农业综合效益和竞争力，确保农业增加值增长3.5%以上。

夯实农业基础。坚守耕地红线，稳定粮食生产，完成土地整治20.6万亩，新建高标准农田34万亩，新增耕地指标1.8万亩。按照“四区合一”〔20〕模式，继续抓好农建综合示范区建设。加强水利设施建设，突出抓好土溪口、白岩滩、石峡子等在建大中型水库建设，加快实施宝石桥水库等中型灌区渠系配套与节水改造，建成万源寨子河水库，积极做好固军、李家梁、大义、斑竹沟等水库前期工作。加强农业科技创新和推广应用，完善农业社会化服务体系。

调优农业结构。优化产品结构，围绕“米袋子”“菜篮子”农产品安全供给，建设一批标准化示范基地和安全食品生产基地；突出发展“3+6”〔21〕优势特色农业，新建现代农业产业基地15万亩；加强农产品质量安全监管，建立生产、加工和流通企业诚信及“红黑名单”制度，开展农产品质量安全示范市县创建。优化产业结构，鼓励工商资本进入农村发展现代种养业，带动特色优势产业标准化、专业化、规模化发展；支持发展休闲观光体验农业、乡村旅游、森林康养产业，促进农村经济多元化发展。

发展加工贸易。坚持“抓二产带一产促三产”的思路，实施新型农业经营主体+农产品精深加工贸易“双培育工程”，推动农业种养加销组织化、信息化、品牌化。抓好知名农产品加工和贸易企业招商，加强在谈企业的跟踪落实，争取2-3家落地建设，推动农产品加工和贸易实现突破性发展。加强对本地名优品牌企业的培育和整合，强化加工和贸易企业与基地合作，推动农业产业转型升级。积极打造农产品区域公共品牌和特色品牌，创建省级以上“三品一标”〔22〕名优品牌15个。

深化农村改革。深入推进农村产权制度改革，细化和落实承包土地“三权分置”〔23〕办法，建立土地流转市场和监管运行中心。鼓励新型经营主体探索融合模式，建立紧密的利益联结机制，让农民有活干有钱赚。多渠道盘活农村资产资源，增加农民财产性收入。稳步推进农村集体经济改革发展，全面深化供销合作社综合改革和国有林场改革。积极推广农业职业经理人制度。扩大激励农业科技人员创新创业试点。做好第三次农业普查。

（四）统筹推进城乡基础建设。我们将抓住全域纳入国家新型城镇化试点机遇，继续加强城乡基础设施建设，着力改善发展条件，推动城乡一体发展，促进市域协调发展。

加强基础设施建设。突出交通设施建设，加快建设达州新机场、营达和巴万高速、达宣和达开快速通道、通川环城产业大道等项目，力争开工建设市绕城高速西段、万源至八台和达渠快速通道、国道210市过境段三期等项目，启动成南达万高铁、巴达和达万铁路扩能改造、达州高铁站、八台山至巴山大峡谷和大竹至百节快速通道、渠江航道升级改造等项目前期工作，全力争取西渝高铁过境达州。抓好能源设施建设，开工建设北外环至经开区输气管线，积极推进宣汉至万源输气管线建设，建成宣汉500千伏输变电工程。加快信息设施建设，突出抓好“宽带中国”示范城市创建、市云计算中心建设，完成“宽带乡村”工程建设。

抓好中心城区建设。围绕构建“双百”区域中心大城市，突出抓好北城滨江、马踏洞、莲花湖、三里坪、长田坝等新区开发，加快建设环城路二期、金南大道、河市大道、南北三号干道南延线，开工建设机场大道、环城路三期和柳家坝、徐家坝、野茅溪、中坝大桥，确保金南大桥、凤凰山隧道建成投用，力争中心城区建成区面积达到90平方公里、人口突破100万。纵深推进缓堵保畅和“五治”“三化”工程，优化完善公交线路、站点和运力结构，新建停车泊位1万个，抓好塔沱、火车站、南坝、川鼓等片区棚改，开工建设快速通道改造、大寨子公园二期、莲花湖湿地生态修复、达川文化体育公园等工程，打造大北街片区商业示范街，建成市第二生活污水处理厂，推进铁路沿线和主城区进出口通道沿线环境综合整治，不断提升城市品质。

统筹城镇新村建设。重点抓好县城新区开发和产业新城建设，同步完善市政设施、公共服务设施，提升人口和产业的承载能力，加快推进Ⅰ型、Ⅱ型小城市建设，积极开展大竹宜居县城、海绵城市试点。深入实施“百镇建设试点行动”，规划建设一批工业强镇、商贸重镇和旅游名镇，提升集聚吸纳能力，确保全市城镇化率提高1.3个百分点。大力实施新村建设“1+5”工程〔24〕和村落改造“5+1”工程〔25〕，建设幸福美丽新村350个，力争20%以上的行政村建成省级“四好村”、30%以上的行政村建成市级“四好村”。

（五）聚力推进脱贫攻坚。今年脱贫攻坚任务异常繁重，我们将以壮士断腕的气魄抓紧抓实，坚决确保实现通川区摘帽、308个贫困村退出、15.2万人脱贫。

着力改善基础条件。严格执行建房标准和补助政策，突出抓好3.78万人易地扶贫搬迁、1.3万农村贫困户危房改造。加快推进农村道路、安全饮水、农网改造、信息网络等基础设施建设，确保308个贫困村通村公路实现硬化、网络实现全覆盖和15.2万脱贫群众安全饮水、用电等问题得到解决。

突出抓好产业扶贫。鼓励引导工商资本进入贫困地区，以大农产品加工和贸易解决农村产业同质化问题，带动贫困地区产业大发展。加强涉农资金整合，用好“三大”扶贫基金，加快培育主导产业。坚持扶智与扶志相结合，加强技能培训和自力更生引导，确保符合条件的计划脱贫户发展1个增收产业或1人实现就业。大力开展电商扶贫，助推贫困地区农特产品走出大山。狠抓旅游扶贫，多渠道增加贫困群众收入。加强金融扶贫，充分发挥扶贫再贷款和小额信贷对产业扶贫的支撑作用。

加强重点人群保障。全面落实农村低保标准线和国家扶贫标准线“两线合一”，确保低保兜底对象不愁吃、不愁穿。加大教育帮扶力度，为家庭经济困难学生提供15年免费教育。深入推进“健康扶贫行动”，认真落实“十免四补助”“八个百分之百”〔26〕卫生扶贫政策，加快推动贫困地区医院软硬件达标。加大救助力度，切实解决因灾致贫返贫、因病致贫返贫、因贫辍学等问题。

完善工作推进机制。严格执行八条超常措施，完善“5+1”帮扶机制，加强专项督促检查，强化县市区主体责任，确保脱贫攻坚组织领导、任务责任等落实到位。持续开展“万企帮万村”行动，支持社会组织参与扶贫开发。深化精准扶贫精准脱贫，完善贫困户动态管理制度，切实做到因户因人施策。强化扶贫资金监管，提高资金使用效益。加强扶贫信息系统建设，适时监测脱贫对象、扶贫项目。完善深化第三方评估，确保真扶贫、真脱贫。

（六）深入推进改革创新开放合作。改革创新是破解难题、推动发展的重要法宝，开放合作是欠发达地区后发赶超的重要途径，我们将认真谋划、深入推进，确保今年取得更大实效。

深化各项重点改革。突出抓好供给侧结构性改革，坚持保优汰劣，继续推进钢铁、煤炭、水泥等行业去产能；着力打通商品房与征拆安置、棚改安置的通道，力争将商品房住宅库存控制在320万平方米以内；全面落实减税降费政策，多措并举降低企业成本。抓好“4+2”重点改革，推动市区责权、城市管理、城市建设、城市公交等按照新的体制机制运行，持续优化行政效能、目标绩效考核。深化国资国企改革，完成市属10类26户国企基础改革工作。推进财税体制改革，健全政府性债务风险防控机制，积极争取国家均衡性财政转移支付，认真落实国家对贫困县、少数民族待遇县、革命老区的财税支持政策。积极参与全省电力体制改革试点。大力支持大竹、渠县撤县设市和符合条件的乡撤乡设镇。密切关注统计制度改革，积极做好“新经济”〔27〕的谋划、发展和统计工作。进一步深化社会保障、教育医疗、资源要素等各项改革，确保取得新的突破。

大力实施创新驱动。全面落实科技创新“十个一批”举措，大力实施制度创新、管理创新、技术创新，引导企业加大研发投入开发新技术、新工艺、新设备、新产品，力争全社会科技创新投入5亿元、高新技术产业总产值达到240亿元以上，确保科技对经济增长的贡献率达到52%。加快推进信息化改造，促进信息化与工业、服务业、制造业等产业互动融合，着力提升经济发展质量。全面落实鼓励创新创业的各项优惠措施，激发民间投资热情，确保民营经济增加值占GDP的比重提高至61%以上。

着力扩大开放合作。加快城际间互联通道建设，主动融入全国全省开放合作大格局。加强国别交流合作，抓好中捷产业园建设，支持玮鑫首饰与俄罗斯、乌克兰合作。加强区域合作，抢抓国家实施川陕革命老区振兴发展规划机遇，联合周边地区共同争取跨区域重大项目及政策。加强与央企省企合作，推进天然气勘探开发和在谈项目的落地建设。加强高等院校、科研院所合作，推进人才培养引进，联合组建研发和检测中心，助推产业转型发展。加强与金融机构合作，争取优惠信贷和低成本融资。加强出口企业及产品培育，抓好经开区、大竹和宣汉园区等出口基地建设。开展“制造业招商突破年”活动，用好“中外知名企业四川行”“渝洽会”等平台，大力实施“达商回引”工程，精心举办全球川商达州行和第二届达商大会，加强产业链和产业集群招商，力争在能源化工、新材料、智能装备制造、电子信息、生物医药、农副产品精深加工等六大重点产业和建筑业招商上取得突破，确保到位资金达到750亿元、制造业招商项目占比达到50%。

（七）持续推进生态文明建设。生态建设和环境保护，既是重要民生，也是发展保障。我们将尊重、顺应和保护自然，以对人民和历史高度负责的态度，努力建设生态文明新家园。

深入实施绿化达州行动。认真落实主体功能区规划，严格划定和控制生态保护红线，切实加强自然保护区、森林公园、地质公园、湿地公园保护建设。启动国家森林城市创建，抓好“沿路、沿河、沿库”生态廊道建设，加快推进环宝石湖生态建设，统筹推进城乡绿化美化。继续抓好天保二期工程、新一轮退耕还林，完成营造林26万亩，新增森林5000公顷。推进废弃矿山复耕和裸露山体整治，加强地质灾害防治，治理水土流失100平方公里，筑牢嘉陵江上游生态屏障。

坚决打好污染防治攻坚战。加大对环保工作的领导和统筹协调力度，建立大气、水、土壤污染防治联席会议制度，打好污染防治“三大战役”。坚持“党政同责”、“一岗双责”，加大环保考核权重，逗硬考核问责。制定并强力实施解决我市环保最直接最突出问题“三年攻坚计划”，集中力量建设一批城镇污水处理、城乡垃圾处理等环保基础设施。加强大气污染防治，开展城市扬尘、工业废气、燃煤锅炉等专项整治，确保空气质量达标率达到75.3%。加强水污染防治，全面落实“河长制”，开展州河、巴河、渠江等重点流域水环境污染和黑臭水体综合整治，确保出境断面水质持续向好。加强土壤污染防治，开展土壤污染详查，抓好污染土壤治理与修复，防控土壤环境风险。加强环境监管执法，始终保持打击环境违法行为的高压态势。

大力发展绿色低碳循环经济。加快发展清洁能源、节能环保装备等绿色产业，支持采购绿色环保产品，扩大节能产品运用。实施循环发展引领计划，推行企业循环式生产、产业循环式组合、园区循环式改造，支持柳池工业园创建省级循环化改造示范园区。抓好工业、交通、建筑等重点领域节能降耗，加快淘汰落后产能和工艺设备，倒逼“两高”行业转型升级。倡导和鼓励绿色生活方式，促进绿色消费、绿色出行、绿色居住成为公众自觉行动。

（八）着力推进民生社会建设。为政之道，民生为本。我们将坚持民生投入只增不减，大力发展社会事业，着力提升社会治理水平，让全市人民共享改革发展成果。

切实保障和改善民生。扎实推进九项民生工程，着力办好全市17件民生大事和中心城区10大民生实事，确保一般公共预算民生支出比例不低于70%。坚持就业优先，妥善安置转业军人和去产能企业职工，加强高校毕业生就业指导，确保城镇新增就业3.4万人。加强重要商品和农产品价格监管，完善社会救助和保障标准与物价上涨挂钩联动机制，保障低收入群众基本生活。推进城乡社保体系建设，加快实施全民参保计划，完善城乡居民基本养老保险制度，整合城乡居民医保，加强社保基金征缴和管理。大力发展老龄、残疾人、慈善、红十字会等事业，加强对农村留守儿童、妇女、老人的关爱服务。

大力发展社会事业。推进义务教育均衡发展，确保达川、大竹通过国家认定。坚持引进和培育相结合，有效整合利用教育资源，做大地方教育总量。推进达一中、达州中学等示范中学改革创新，打造一批秦巴区域名校。加快西南职教园区建设，推进城区中职学校搬迁入园，整合职业教育资源，支持与省内外大学联合创办高职院校。推进四川文理学院转型发展应用型大学和达州职业技术学院创建优质专科高职院校。支持学前教育、继续教育发展。深入实施健康达州行动，加强重大传染病防治，完善分级诊疗制度，抓好与华西医院合作办医，提升达州医疗水准。大力发展体育健身运动，着力增强人民体质。深入实施公共文化惠民工程和“1223”巴文化保护工程〔28〕，支持县级文化馆、图书馆、博物馆、科技馆、体育馆、档案馆、工青妇活动中心、残疾人服务中心建设。鼓励文艺创作，推动文艺事业繁荣发展。认真做好民族、宗教、统计、气象、保密、档案、地方志、对台、外事、侨务等工作，促进社会全面进步。

提升社会治理水平。完善基层治理体系，健全基层综合服务管理平台，增强村（社区）自治功能，提高基层社会治理科学化、法治化水平。发挥人民团体作用，支持参与社会治理。深化“法治”达州建设，推进依法治市“十大行动”，广泛开展“七五”普法，做好法律援助和社区矫正工作，加强司法基层基础和公共法律服务体系建设。健全社会信用体系，加强政务、商务、社会诚信和司法公信建设。依法分类处理信访诉求，落实社会稳定风险评估，推进矛盾纠纷多元化解。加强食品药品监管，以更为浩大的声势和更加有力的措施严厉打击食品药品违法行为，确保人民群众饮食用药安全。落实安全生产责任，加强防灾减灾，坚决遏制重特大事故发生。强化网络和危机管理，提高舆情引导和突发事件应急处置能力。深化“平安达州”建设，加快城市“天网”工程和农村“雪亮”工程〔29〕建设，完善立体化治安防控体系，依法打击各类违法犯罪，维护公共安全和社会稳定。加强国防动员、国防教育和国防后备力量建设，积极做好双拥共建、人民防空等工作，巩固军政军民团结。

四、加强政府自身建设

各位代表！新形势新任务对政府工作提出了新的更高要求，在新的一年里，我们将始终坚持敬重人民、敬畏法纪，始终坚持科学理政、依法行政、民主施政、高效治政、廉洁从政，全面加强政府自身建设，努力建设人民满意政府。

坚定政治方向。牢固树立“四个意识”〔30〕，严守政治纪律政治规矩，在政治上思想上行动上始终与中央和省委保持高度一致。坚决维护市委总揽全局、协调各方的核心领导作用，始终与市委同心同德、同向发力，切实把市委的决策部署到位、执行到位、落实到位，坚决维护政治安全特别是政权安全和制度安全，实现好、维护好、发展好广大人民群众的根本利益。

严格依法行政。忠实履行宪法和法律赋予的职责，做到尊法学法守法用法。深化法治政府建设，健全依法科学民主决策机制，规范权力运行和公正文明执法，加强政府立法工作，改进行政复议和行政应诉工作。推进政务公开透明，及时回应社会关切。主动接受市人大、市政协监督，加强与民主党派的联系，重视司法、舆论和公众监督，广泛听取和吸纳各方意见。

提升行政效能。深化“放管服”改革，以清单管理推动减权放权，加强事中事后监管，实现“双随机一公开”〔31〕监管全覆盖，重点在政府运行效率、行权平台规范运行、公共资源交易场外代理、政务服务前置事项上下功夫，让市场和社会活而有序。提升政务信息化水平，大力实施“互联网+政务服务”，推动网上与实体政务大厅融合发展，逐步实现“一号申请、一窗受理、一网通办”。开展减证便民行动，清理取消不合法不合理的证明和手续，让群众办事更加便捷。

敢于担当担责。严格落实工作岗位责任制、督查问责制和第三方绩效评价，坚决整肃庸政懒政怠政行为，确保政府工作掷地有声、落地生根。健全容错纠错机制，保护干事创业激情，为担当者担当，为负责者负责，让广大干部想干事敢干事干成事。坚持直面矛盾敢于担当，优化服务主动担当，依法依规善于担当，认真负责真情担当，以担当担责精神应对新挑战、争创新业绩。

加强廉政建设。严格落实党风廉政建设责任制，坚决把中央和省、市委从严治党、从严治政各项规定贯彻到政府工作各环节。完善行政权力依法规范公开运行机制，深化廉政风险防控，扎紧制度笼子。加强行政监察、审计监督，严厉查处违法违纪行为，坚定不移惩治腐败。持续深入改进作风，巩固“三严三实”“两学一做”成果，让人民群众从政府工作中感受到清风正气。

各位代表！新的一年开启新的希望，新的征程承载新的梦想。让我们更加紧密地团结在以习近平同志为核心的党中央周围，在省委省政府和市委的坚强领导下，开拓进取，扎实工作，奋力推进同步全面小康和幸福美丽达州建设，以优异成绩迎接党的十九大和省第十一次党代会胜利召开！
